# Dont’s for Consumptives

**Published:** 1896-02

**Authors:** 


DONT'S FOR CONSUMPTIVES; OR, THE SCIENTIFIC MANAGEMENT OF PULMONARY TUBERCULOSIS, is the title of a book, which, under the authorship of Dr. Charles Wilson Ingraham, will soon (about February 10th) be issued by the Medical Reporter Publishing Co., of Rochester, N. Y. The book will be printed on 72-pound antique book paper, bound in cloth (imitation morocco), with title in good leaf. Price, $1.75.

The complete work of thirty-five chapters is devoted exclusively to the general management of pulmonary invalids, no reference whatever being made to drug treatments.

The object of the author is to supply the physician with a practical work, and at the same time, by eliminating technical terms, reduce the text within the easy comprehension of the intelligent patient. The author claims that "a good understanding of his condition is the best remedy for the consumptive." With this book in the hands of his patient the physician will be relieved of a multitude of details which attach to the successful management of such cases. Special attention has been given those chapters pertaining to the destruction of tubercular infection.

